# Predicting Fracture Risk in Patients with Metastatic Bone Disease of the Femur: A Pictorial Review Using Three Different Techniques

**DOI:** 10.1155/2021/5591715

**Published:** 2021-06-16

**Authors:** Shannon M. Kaupp, Kenneth A. Mann, Mark A. Miller, Timothy A. Damron

**Affiliations:** SUNY Upstate Medical University, Department of Orthopedic Surgery, 750 East Adams Street, Syracuse, NY 13210, USA

## Abstract

One of the key roles of an orthopedic surgeon treating metastatic bone disease (MBD) is fracture risk prediction. Current widely used impending fracture risk tools such as Mirels scoring lack specificity. Two newer methods of fracture risk prediction, CT-based structural rigidity analysis (CTRA) and finite element analysis (FEA), have each been shown to be more accurate than Mirels. This case series illustrates comparative Mirels, CTRA, and FEA for 8 femurs in 7 subjects. These cases were selected from a much larger data set to portray examples of true positives, true negatives, false positives, and false negatives as defined by CTRA relative to the fracture outcome. Case illustrations demonstrate comparative Mirels and FEA. This series illustrates the use, efficacy, and limitations of these tools. As all current tools have limitations, further work is needed in refining and developing fracture risk prediction.

## 1. Introduction

Bone is the third most common site for cancer metastases after lung and liver [1]. Metastatic bone disease (MBD) has become an increasingly prevalent condition as the population of patients over forty increases and the longevity for patients with cancer continues to improve [2]. Patients with MBD often present with bone pain, impaired mobility, and impending or actual pathologic fracture [1, 3]. The challenge of impending fracture lies in determining which warrants prophylactic treatment, particularly operative stabilization. Early clinical and radiographic attempts to define impending pathologic fracture, including Mirels score [4], show poor specificity and variable sensitivity. Strict adherence to Mirels score and recommendations for prophylactic stabilization would lead to unnecessary surgeries [5]. Numerous other tools suffer the same limitations [6–8].

Two more recently utilized techniques, CT-based structural rigidity analysis (CTRA) and finite element analysis (FEA), have reported improved accuracy in predicting fracture in long bones, particularly the proximal femur [5, 9–14]. Although not yet widely used as a clinical tool, CTRA accounts for changes in bone density and bone geometry due to the presence of MBD lesions. With CTRA analysis, the rigidity of the bone is calculated using CT data for the bone, and a predetermined loss of rigidity threshold is used to create a binary outcome of increased risk or not increased risk of fracture [13, 15]. CTRA has been shown to be superior to Mirels score in terms of predicting fracture risk in terms of sensitivity, specificity, positive predictive value, and negative predictive value [5]. With FEA, a computer model of the femur with the lesion is created from the CT scan with material properties of each element of the model assigned based on bone density. Force to failure can be calculated by applying loads to the model simulating daily activities including standing, walking, and stair ascent [9–11]. FEA has also been shown to be a superior method when compared to Mirels score and expert opinion [9, 16].

Although CTRA and FEA have been reported to be more accurate than Mirels score when assessing fracture risk, neither CTRA nor FEA has been clearly demonstrated to be the superior technique. The primary purpose of this pictorial essay is to demonstrate the comparative and complementary use of Mirels, CTRA, and FEA in the setting of MBD in cases where all three tests were performed. A secondary goal is to familiarize clinicians with these techniques. With CTRA as the reference test, cases of true positives, false positives, false negatives, and true negatives are illustrated. This is the first report of its kind for MBD.

## 2. Materials and Methods

This research was deemed exempt by the Institutional Review Board at SUNY Upstate Medical University (UMU). Cases were extracted from SUNY UMU patients enrolled in a prospective Musculoskeletal Tumor Society (MSTS) sponsored study. The MSTS multi-institutional study has enrolled MBD patients since July 2008 and has continued through 2021. The primary purpose was to establish the efficacy of fracture prediction using CTRA. Patients from the MSTS CTRA study at UMU have also been analyzed by FEA, and those patients from 2008 to 2012 have been previously reported [11].

Initial enrollment in the MSTS study included all long bones and involved assigning a Mirels score and obtaining X-ray imaging and a CT scan with phantom. The CT scans with phantom were used for initial CTRA and subsequent FEA. Patients who did not undergo prophylactic stabilization surgery were followed at approximately 4-month intervals to assess changes in their condition and radiographic lesional progression.

**Figure 1:**
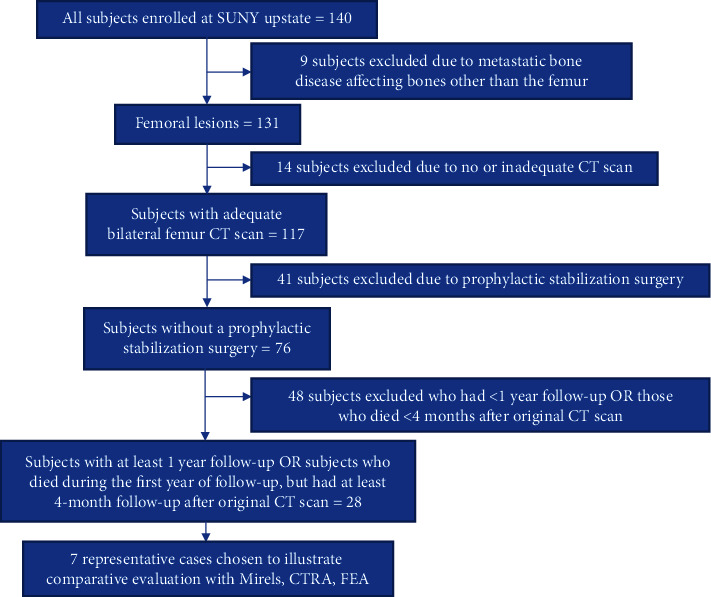
Subject exclusion flow chart.

Enrollment criteria for the current report were patients enrolled at SUNY UMU with MBD lesions of the femur for which Mirels, CTRA, and FEA had been performed and with follow-up available to time of fracture or death or a minimum 12-month follow-up. Excluded criteria were patients who had nonfemoral lesions analyzed by CTRA, did not have all three prediction outcomes available, had undergone prophylactic stabilization, or had less than 12-month follow-up without fracture or death. Patients who died within 12 months were included if there was a minimum 4-month follow-up. For the cases with patients who went on to fracture, we eliminated any case thought to have potentially been caused by an osteoporosis. Additional reasons for exclusion included elective discontinuation from the study, lack of X-ray images, and incomplete medical records (Figure 1). From the final group, we selected the most illustrative true positive, false positive, false negative, and true negative cases using CTRA as the reference test.

Many of the subjects had bilateral femoral lesions at presentation. However, case presentations were selected based upon the criteria depicted in Figure 1. Hence, for the final selected cases, details and figures for the contralateral femur are only discussed briefly where applicable.

A Mirels score was assigned to each subject by a single orthopedic oncologist (TAD) using information from both the initial visit and imaging. Scoring was determined based on the X-ray imaging, not the CT scans. Mirels score consisted of four components (size, location, tumor type, and pain level), with 1–3 points assigned to each component. A total score of up to 12 points was possible [4, 17].

Computed tomography (CT) scans were performed with a hydroxyapatite phantom (0, 500, 1,000 mg/cc HA or 0, 750, 1,500 mg/cc HA) in view to calibrate the mineral equivalent density of the patient's bone [11]. The phantom was used for both CTRA and FEA to correlate the degree of X-ray attenuation with an accurate bone mineral density (BMD) measurement. CTRA is a technique that estimates a bone's fracture threshold through loss of bone rigidity (in this case the femur). Of note, CTRA has traditionally been calculated using straight beam theory, but more recently a method of curved CTRA has been developed [13]. When referencing CTRA within this manuscript, all subjects were assessed using traditional straight beam theory. The likelihood of fracture is based on axial (EA), bending (EI), and torsional (GJ) bone rigidity at the weakest CT cross section through the lesion. Bone rigidity is an indirect measure of the bone's resistance to fracture when a force is applied. CTRA calculates the elastic modulus (stiffness, E or G) of each pixel in the bone cross section using the local bone mineral density. The product of the pixel modulus (E or G) and section properties (A, I, J) are then calculated. A more detailed description of this technique has been described in the literature previously [18]. CT scans obtained locally were electronically and securely transferred to Beth Israel Deaconess Biomechanics Laboratory, Harvard University, Boston, Massachusetts, for analysis. As a research tool in development, CTRA was not always available for clinical decision making.

FEA was not part of any clinical decision making but rather was performed at a later time and as a research tool only. The FEA data was not available concurrently with the patient evaluation and therefore played no role in a patient's treatment plan. For the analysis with FEA, Mimics software was used to create voxel-based finite element meshes based on the CT scan of the femur with lesion. Material properties were assigned to each finite element based on the mineral equivalent density using the CT scan gray scale. Femoral head and abductor loads were applied to the model consistently with axial load, level walking, and stair ascent [11]. The force needed to fracture the femur was calculated for each loading condition. The risk of fracture (ROF) was calculated as 3 times the body weight (3 BW) divided by the predicted fracture load. If the ROF was greater than 1, fracture was predicted. The loading conditions used in this study (femoral head load and abductor load) were sufficient to assess fracture risk for proximal and midshaft femoral lesions only but were not used sufficiently to assess cases with distal femoral lesions.

## 3. Case Summaries

Cases are grouped according to the category of fracture prediction using CTRA as the primary technique relative to the true fracture outcome (Table 1). Mirels and FEA results are also presented for comparison. In only one of the cases, where both CTRA and FEA were available, was there a discrepancy in the findings (Case 7, left femur).

### 3.1. CTRA True Positive: Case #1 (Right Femur)

**Figure 2:**
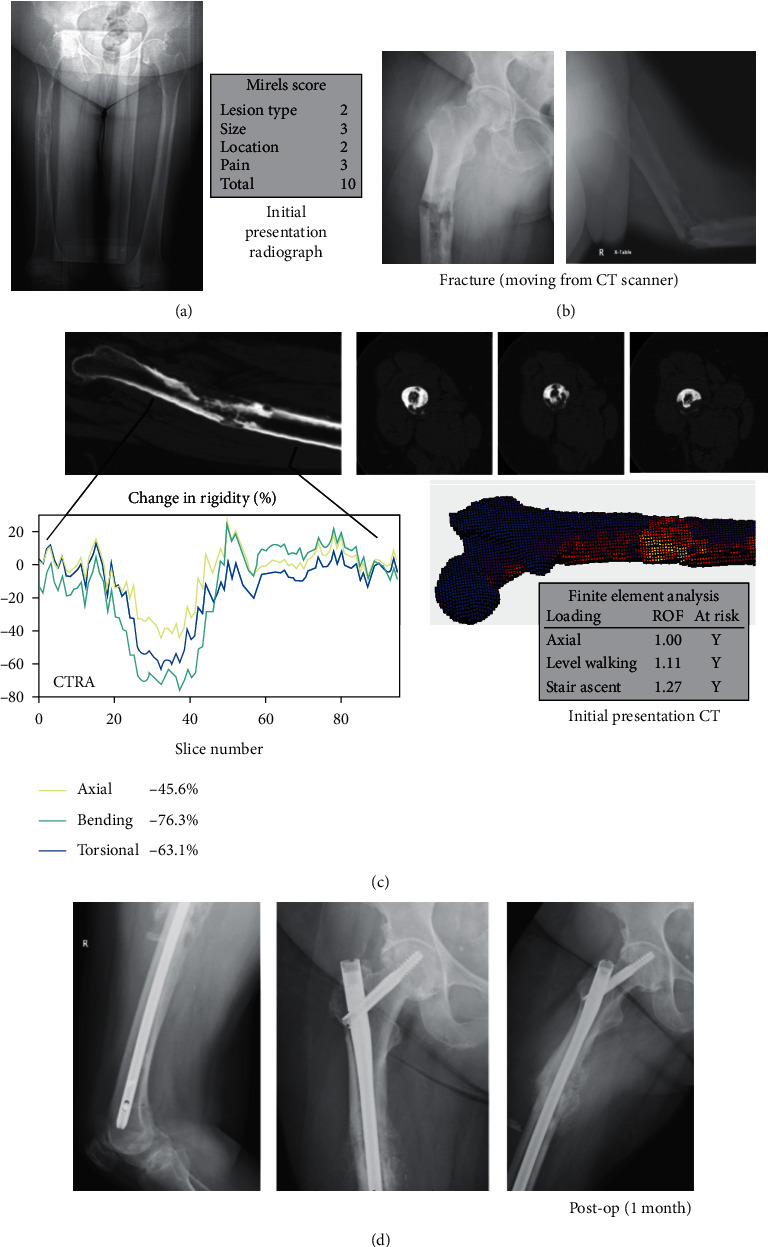
True positive (CTRA+/Fx+) (Case 1, R femur). (a) This is a 68-year-old female with metastatic breast cancer showing an osteolytic lesion in the right femoral diaphysis on X-ray imaging, (b) who, while moving from the CT scanner, fractured her right femur. (c) CTRA and FEA analysis both predicted fracture in this patient. (d) She was admitted and taken for surgical fixation of the right femur.

A 68-year-old female presented with excruciating right thigh pain associated with a femoral metastatic lesion (Figure 2). She had a history of triple negative (ER-, PR-, Her2-) left breast infiltrating ductal carcinoma treated six years ago with bilateral mastectomies, radiation therapy, and multiple chemotherapeutic agents. Shortly following her office visit, while moving from the CT scanner, she fractured the proximal right femoral diaphysis. The following day, she underwent open biopsy followed by ORIF with an intramedullary reconstruction nail. Post-op EBRT was given. For the right femur, her Mirels score was 10, and both CTRA and FEA suggested that she was at increased fracture risk based on the prefracture CT. Due to the chronology, both the CTRA and FEA were calculated post hoc. Over the next year, new lesions were discovered in the left femur, but since the patient was asymptomatic, no surgical intervention occurred.

### 3.2. CTRA True Positive: Case #2 (Right Femur)

**Figure 3:**
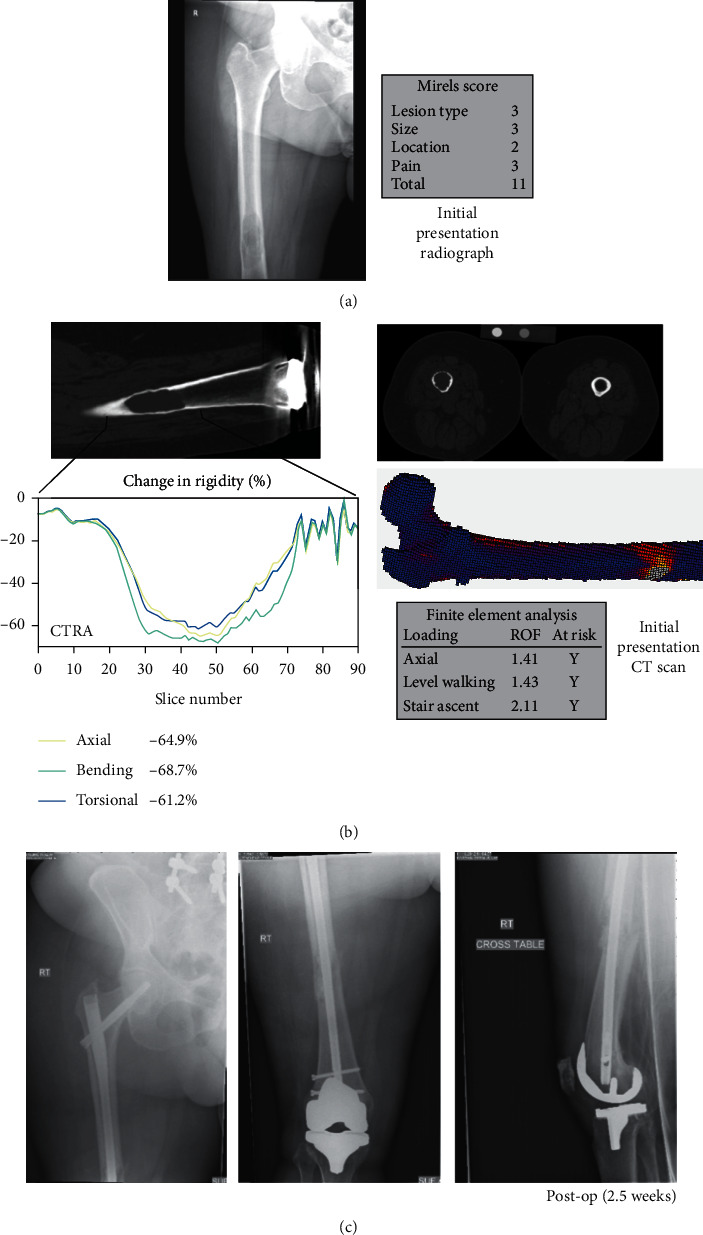
True positive (CTRA+/Fx+) (Case 2, R femur). (a) This is a 77-year-old female with metastatic renal cell carcinoma to the right femoral diaphysis. (b) CTRA and FEA both predicted fracture for this subject. (c) The patient was admitted to the hospital on the day of her initial appointment and twisted her leg in bed, likely causing a fracture. When taken to the OR for prophylactic stabilization, the patient was noted to have a fracture and thus was treated with ORIF.

A 77-year-old female with a history of renal cell carcinoma status following partial nephrectomy three years ago presented due to two years of progressive right thigh pain no longer responsive to oxycodone and causing difficulty with ambulation (Figure 3). A large right femoral diaphyseal lytic lesion was interpreted as an impending fracture. Mirels score was 11, and both CTRA and FEA suggested increased fracture risk.

She was directly admitted from the office due to poor health and need for additional malignancy staging and for interventional radiology biopsy prior to planned prophylactic stabilization. Additional metastatic lesions were noted on the chest wall and right humerus. Shortly after admission, she twisted her right lower extremity in bed resulting in increased right thigh pain, causing pathologic fracture through the lesion. Interventional radiology CT guided biopsy confirmed metastatic renal cell carcinoma. Preoperative embolization the following day was followed by subsequent ORIF with a femoral reconstruction nail on the next day.

### 3.3. CTRA False Positive: Case #3 (Right Femur)

**Figure 4:**
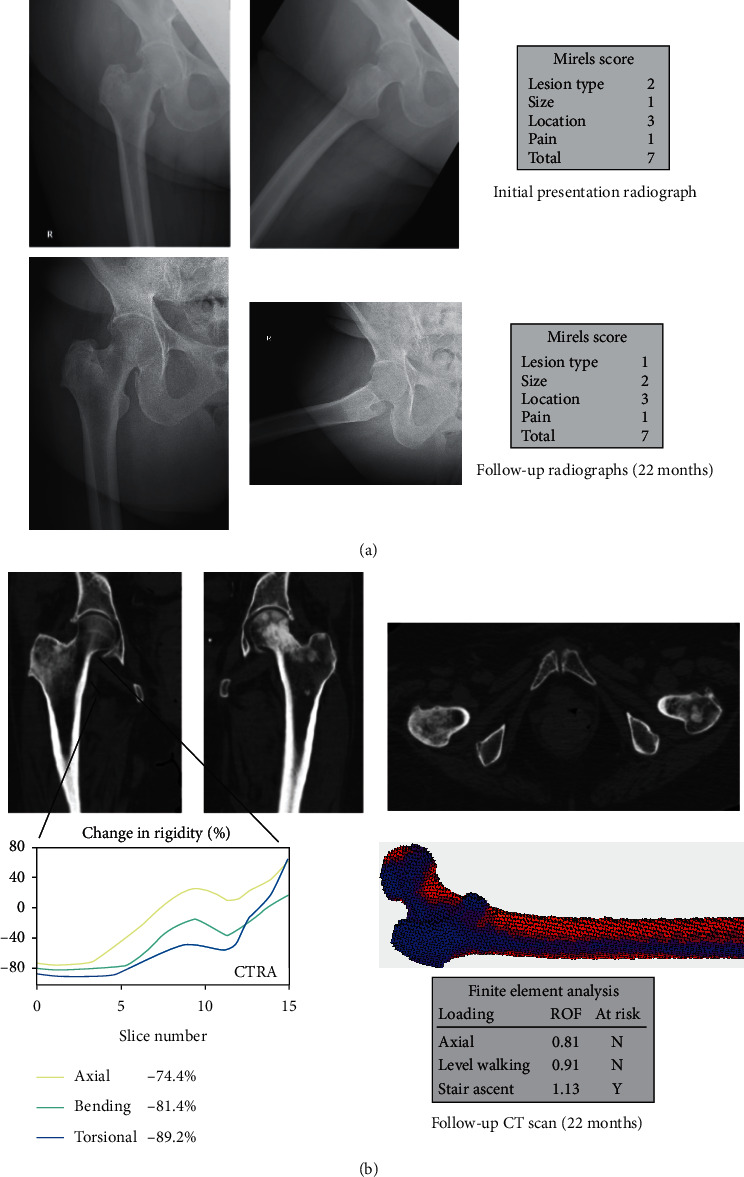
False positive (CTRA+/Fx-) (Case 3, R femur). (a) This is a 59-year-old female with metastatic breast cancer showing a mixed osteoblastic and osteolytic lesion in the right femoral neck. (b) Both CTRA and FEA predicted that the subject would fracture; however, due to a low Mirels' score and mild pain, she did not undergo prophylactic stabilization surgery.

A 59-year-old female originally presented with persistent mild right hip pain (Figure 4). She had a history of breast cancer treated with lumpectomy, lymph node dissection, EBRT, and multiple chemotherapy agents 7 years ago. At presentation, she had known metastatic lesions in the sternum and L4 vertebrae. Imaging revealed a right greater trochanter lesion. Mirels score was 7, and prophylactic stabilization was not recommended. Instead, she underwent right proximal femoral EBRT.

Two years later, she returned with bilateral femoral metastatic lesions. The Mirels score for her right femur was unchanged at 7. However, both CTRA and FEA suggested increased fracture risk. Prophylactic right femoral stabilization was discussed due to the positive CTRA findings but ultimately was not recommended due to the low Mirels score and mild pain. Two years later, she returned with severe right hip pain associated with advanced osteoarthritis and a nondisplaced acetabular dome pathologic fracture through a periacetabular lytic lesion. However, there was no proximal femur fracture.

Images shown are for the right femur. Of note, for the left side, a Mirels score of 9 (2 for pain, 3 for size, 1 for lesional characteristics, and 3 for location) was followed by CTRA showing no increased risk of fracture. The left hip was treated with EBRT but no operative intervention.

### 3.4. CTRA False Positive: Case #4 (Right Femur)

**Figure 5:**
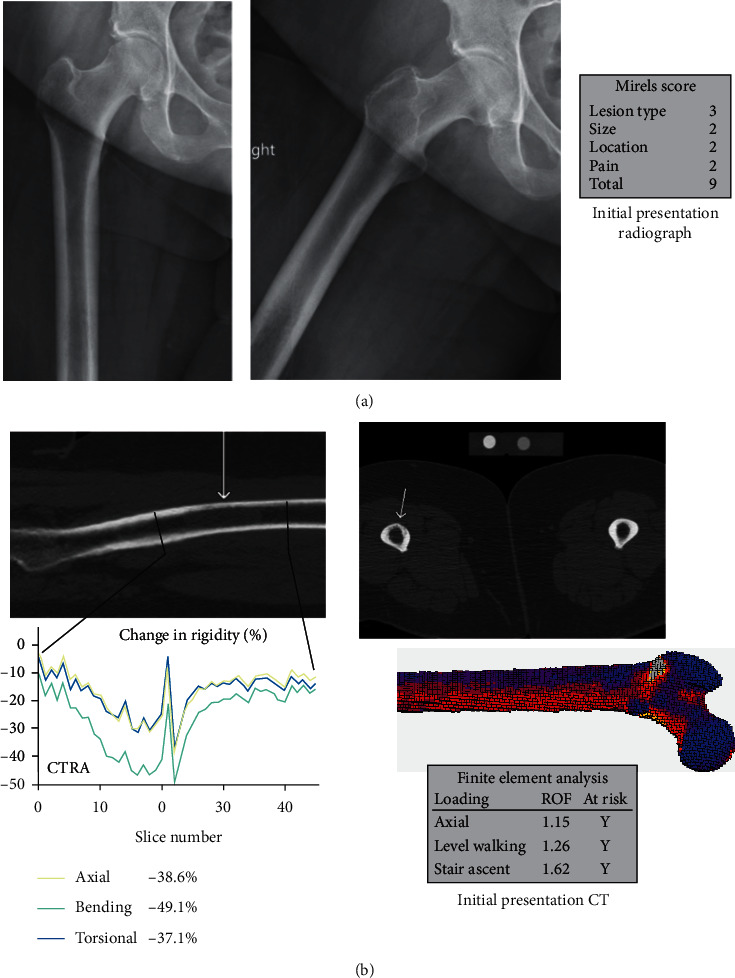
False positive (CTRA+/Fx-) (Case 4, R femur). (a) This is a 68-year-old female with metastatic neuroendocrine carcinoma demonstrating an osteolytic lesion in the right femoral diaphysis. (b) Both CTRA and FEA predicted that the patient would fracture; however, she was treated nonoperatively with EBRT and did not fracture over the course of her one-year follow-up.

A 68-year-old female presented to the ER with pathologic right humerus fracture and progressive right lower extremity pain rendering her nonambulatory (Figure 5). She had been diagnosed with metastatic neuroendocrine carcinoma one month ago. At that time, PET/CT scan showed metastatic lesions in the lymph nodes, liver, and multiple bones (right clavicle, right scapula, right humerus, left iliac crest, and bilateral femurs). For the right femoral midshaft diaphyseal lesion, Mirels score was 9, and both CTRA and FEA suggested increased fracture risk. The humerus was treated by ORIF. The right femoral lesion was treated nonoperatively with EBRT, and despite regaining an active lifestyle, she did not fracture over the subsequent year.

Images shown are for the right femur. Of note, the left femur demonstrated a distal metaphyseal lesion, for which Mirels was 9 and CTRA suggested no increased fracture risk. FEA was not run on the right femur because the model is not designed for distal femoral lesions. No fracture occurred over the year after EBRT.

### 3.5. CTRA False Negative: Case #5 (Left Femur)

A 63-year-old male presented with pain associated with a pathologic humeral fracture. Past medical history was significant for metastatic renal cell carcinoma to lung, lymph nodes, and bone (right ilium, right humerus, bilateral femurs). Due to the newly diagnosed bone metastases, he underwent dedicated X-ray and CT imaging of the bilateral femurs.

**Figure 6:**
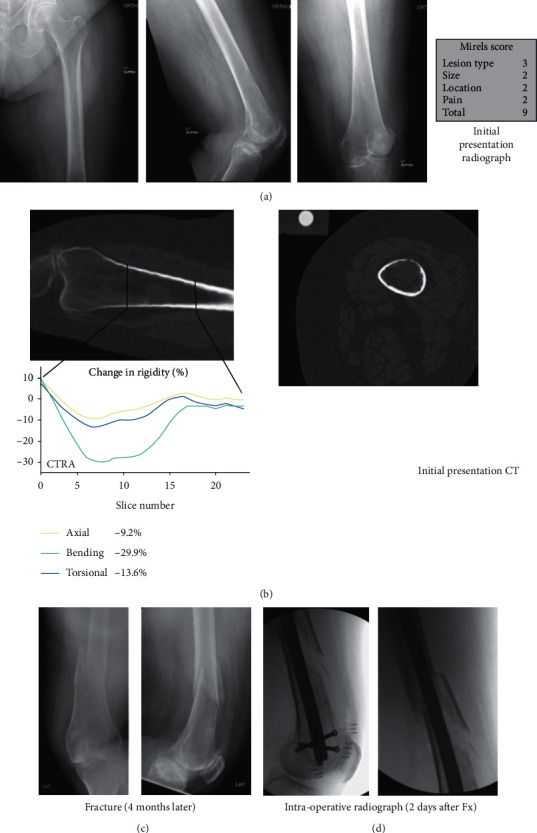
False negative (CTRA-/Fx+) (Case 5, L femur). (a) This is a 63-year-old male with metastatic renal cell carcinoma in the distal femoral diaphysis. (b) CTRA did not predict fracture in this patient, and FEA was not conducted due to the distal nature of this lesion. (c) The subject had a fall resulting in a fracture of the left femur and (d) underwent operative fixation.

A lytic left distal femur lesion was identified (Figure 6). Mirels score was 9, and CTRA did not predict increased fracture risk. FEA was not run on the left femur because the model is not designed for distal femoral lesions. No specific treatment was recommended. Four months later, the patient fell and was found to have a closed pathological fracture of the left femoral shaft and underwent operative fixation with an intramedullary reconstruction nail. He went on to have a complicated hospital course with UTI, bilateral pleural effusions, and right-sided paralysis associated with hemorrhagic stroke due to metastatic brain lesions, leading ultimately to death. It is possible that prior prophylactic stabilization of the femur could have prevented this outcome. Pathologic fractures can be devastating complications of MBD, thus making accurate fracture prediction essential. However, prophylactic stabilization surgery comes with its own inherent risks to the patient, so weighing the risks and benefits for each patient is crucial.

All figures are for the left femur. For the right femur, CTRA was not run and a Mirels score was not assigned because a lesion was not recognized on X-ray imaging. It was only with CT imaging that the right side was noted to contain a metastatic lesion. Post hoc FEA was run for the right femur and predicted fracture. No prophylactic stabilization was recommended. Two months prior to the left femur fracture, he fell down some stairs and incurred both a right hip and right humeral fracture. He underwent hip hemiarthroplasty and ORIF humerus.

### 3.6. CTRA True Negative: Case #6 (Right Femur)

**Figure 7:**
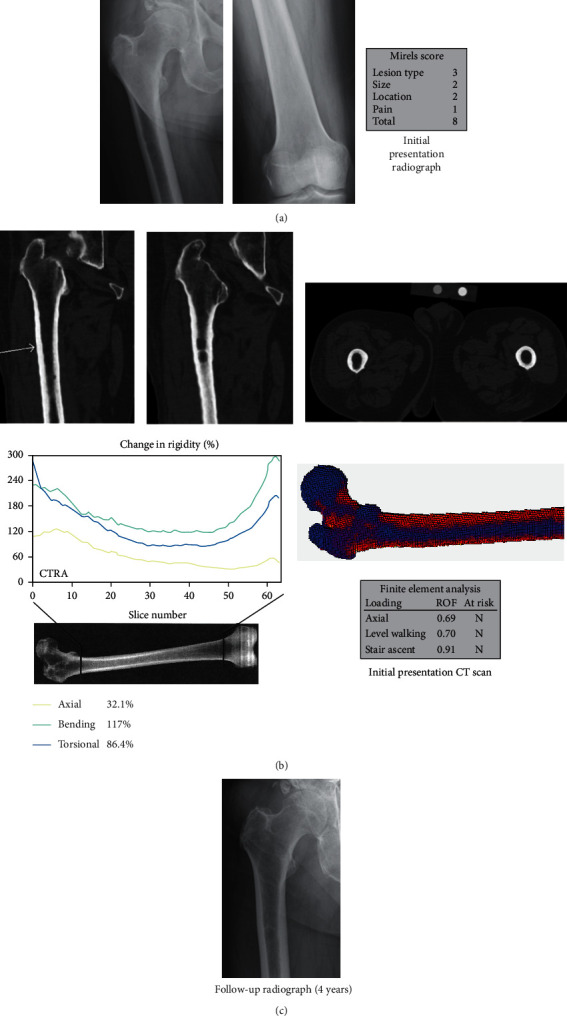
True negative (CTRA-/Fx-) (Case 6, R femur). (a) This is a 79-year-old male with a 12-year history of multiple myeloma demonstrating osteolytic lesions within the right subtrochanteric femur. (b) CTRA and FEA were both run and did not predict fracture for this subject. (c) The patient was followed for several years, and follow-up imaging at four years demonstrates minimal change to the subtrochanteric lesion.

A 79-year-old male with a history of multiple myeloma diagnosed 12 years ago presented with a 9-month history of aching pain in his right pelvis that began suddenly while doing minor work in his yard (Figure 7). Both CT and X-ray showed right iliac pathologic fracture and numerous bilateral femoral lytic lesions. For his right femur, Mirels score was 8, and neither CTRA nor FEA suggested increased fracture risk. Biopsy of the iliac bone confirmed the lesions to be due to multiple myeloma. Treatment consisted of pelvic EBRT and systemic immunotherapy. At 4-year follow-up, no femoral fracture occurred. The patient had no left lower extremity pain, and therefore neither Mirels nor CTRA was assessed.

### 3.7. CTRA True Negative: Case #7 (Left and Right Femurs)

**Figure 8:**
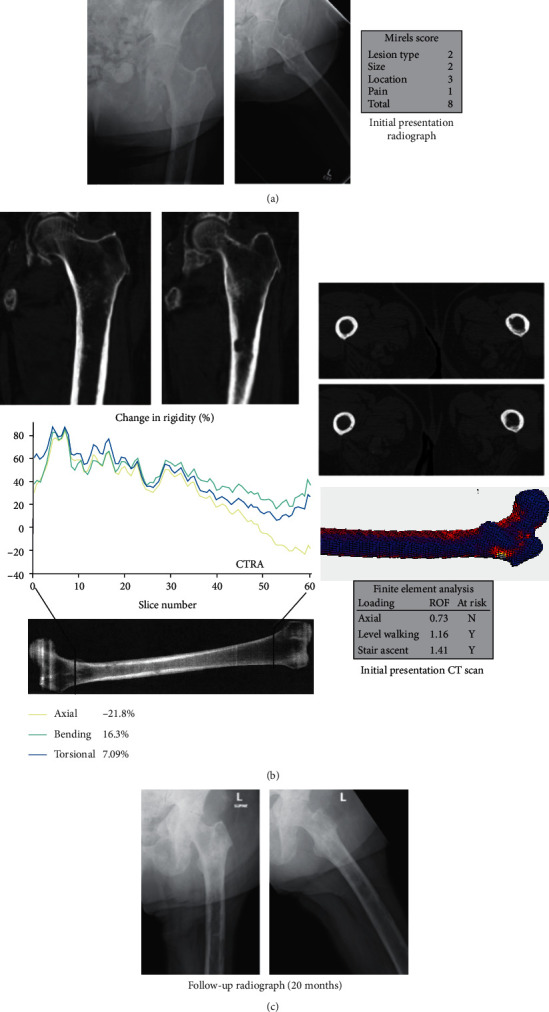
True negative (CTRA-/Fx-) (Case 7, L femur). (a) This is a 72-year-old female with breast cancer and extensive MBD showing a mixed osteoblastic and osteolytic lesion in the left femoral diaphysis. (b) While CTRA predicted that the subject would not fracture, FEA did predict fracture. (c) She was followed for two years without evidence of fracture. Imaging taken at 20 months demonstrates MBD without a fracture.

**Figure 9:**
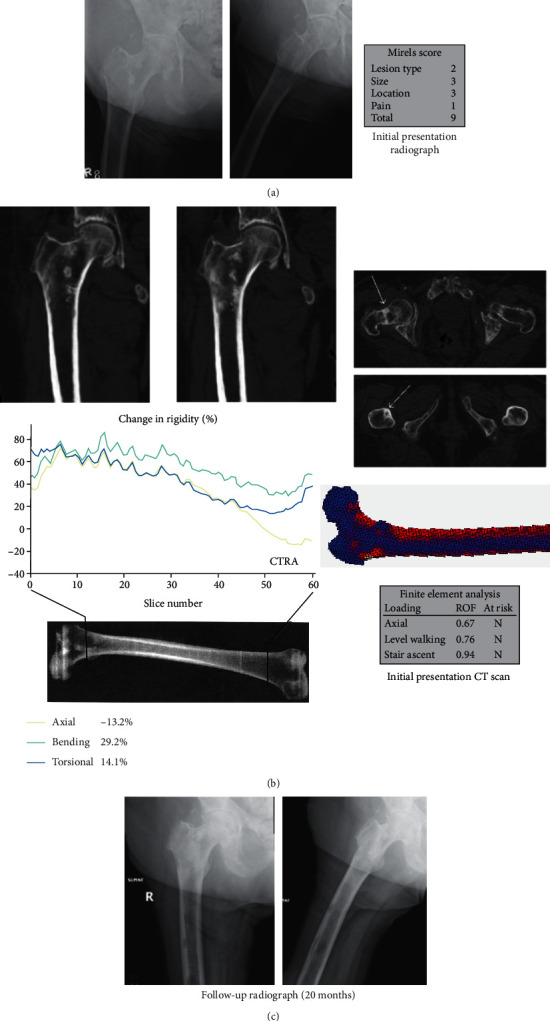
True negative (CTRA-/Fx-) (Case 7, R femur). This figure is for the same subject in Figure 8 but now depicts the right femur. (a) Initial X-ray imaging shows a mixed osteoblastic and osteolytic lesion in the intertrochanteric region of the right femur. (b) Both CTRA and FEA were run and neither predicted the subject to fracture. (c) Imaging taken 20 months after her initial evaluation demonstrates progression of her MBD but without fracture.

A 72-year-old female with a history of MBD due to breast cancer presented with a 7-month history of right upper extremity and left groin pain after a fall on ice. Multiple identified sites of MBD included bilateral femurs (Figures 8 and 9), bilateral humeri, anterior and posterior ribs, iliac bones, and sacrum, as well as diffuse spine and skull lesions.

The left proximal femoral diaphyseal lesion Mirels was 8, and CTRA did not suggest increased fracture risk. However, FEA suggested increased fracture risk. Prophylactic stabilization was not recommended. As she did not go on to fracture through 2-year follow-up, CTRA was a true negative while FEA was a false positive. This was the only case in our series where the findings for CTRA and FEA differed.

The right intertrochanteric femoral lesion Mirels was 9, and both CTRA and FEA did not suggest increased fracture risk. Prophylactic stabilization was not recommended. As noted above, no fracture occurred through 2-year follow-up.

## 4. Results

The above seven cases depict true positive, true negative, false positive, and false negative examples in respect of fracture outcome as predicted by CTRA. Since they were hand-selected for this case series, instead of randomly chosen, the results of the cases cannot be combined and instead are unique examples of CTRA prediction with outcome. Table 1 shows a summary of each of the cases. Case 5 is unique in that it is the only case out of the 140 we analyzed where CTRA had not predicted fracture, but the patient indeed went on to fracture. As shown in Table 2, literature has reported CTRA sensitivity at 100%, so it is rare to find a false negative CTRA example. This is also why each category has two cases whereas there is only one case representing the false negative category.

## 5. Discussion

Fracture risk prediction in MBD is important to identify patients who will benefit from prophylactic stabilization by avoiding problems inherent to ORIF and separate them from those for whom operative intervention is unnecessary, poses unneeded risks, and increases cost of care [19–24]. Although one of the goals of prophylactic stabilization is to prevent the morbidity of fracture, other purported advantages include pain relief and improved function.

Numerous historic clinical and radiologic based techniques have been used for fracture risk prediction in MBD [16, 25]. Mirels scoring system, based on plain radiographs and clinical findings, is widely taught and used, but newer CT-based techniques including CTRA and FEA have shown more accurate risk prediction [5, 9–13]. The cases presented in this pictorial review illustrate the nuances of utilization of all three (Mirels, CTRA, FEA) in individual cases.

### 5.1. Mirels Clinical Scoring

The Mirels score was originally developed and reported in 1989 [4]. Scoring ranges from four to twelve depending on the patient's reported pain level, the lesional characteristics (lytic/blastic/mixed), the size of the lesion, and the location of the bone lesion determined by X-ray imaging. Based upon the recommendation of Mirels, lesions deemed to have a total score of nine or more were considered to have an impending fracture, and prophylactic stabilization was recommended [4, 14]. Mirels found a sensitivity of 81% and specificity of 94% when doing a retrospective analysis of 38 patients (78 lesions) on bones in both the upper and lower extremities using this criterion. In his analysis, he found that, among the four score components, the patient's pain level and lesion size were the most predictive of impending fracture [4]. However, it is important to note that Mirels developed the scoring system from a single training data set, with the goal of achieving the best combination of sensitivity and specificity. Ideally, this scoring system would have been applied to an independent test (or validation) data set.

Further application of the Mirels scoring system by other authors has not revealed results as promising as the original Mirels series, particularly with regard to specificity. Five more recent manuscripts have reported a sensitivity in the range of 67–100% but a specificity in the range of 13–50% [5, 11, 12, 26, 27] using Mirels scoring for femoral lesions, suggesting that the Mirels score may overpredict fracture risk among patients with MBD. This is an important consideration when using the Mirels scoring system clinically to identify patients who may need prophylactic stabilization surgery.

In the current pictorial review series, utilizing the suggested Mirels score of nine points or more as the criterion for prophylactic stabilization, there were three true positives, three true negatives, two false positives, and no false negatives relative to the actual clinical outcome (fracture vs no fracture). Although the purpose of this study was not to analyze the predictive capability of the Mirels scoring system given the small number of cases, these cases do illustrate the high sensitivity (100%) and modest specificity (60%) of Mirels scoring. The Mirels scoring system remains widely used by physicians, but, due to its low specificity and poor positive predictive value, relying purely on Mirels scoring could lead to many unnecessary prophylactic stabilization surgeries [14, 16, 28].

### 5.2. Computed Tomography Rigidity Analysis (CTRA)

CTRA was developed in an effort to improve upon the Mirels scoring system through identification of bones that may be at an increased risk of fracture due to metastatic lesions. CTRA utilizes three-dimensional CT imaging sets of bone with metastatic lesions, in contrast to Mirels scoring which is based in part upon measures from two-dimensional radiographs. The geometry of the lesion is more fully characterized with CTRA, and the distribution of bone density is also determined. By incorporating the geometry of the bone and the density together, bone rigidity (axial, bending, and torsional) can be calculated. CT scan images are obtained for the bilateral femurs, and CTRA is performed on the involved femur as well as the contralateral femur, which serves as a control. If the femur rigidity has been reduced by 33% or more compared to the contralateral femur (without MBD), it is considered to be at a high risk of fracture [12, 16]. In cases where the contralateral femur cannot be used as a control, such as bilateral MBD, CTRA uses standardized age and sex matched femurs as the control. The use of the patient's contralateral femur is the preferred method and functions as a built-in control when calculating the loss of rigidity.

Several investigators have assessed the sensitivity and specificity of CTRA to determine its usefulness as a clinical tool [5, 10, 12, 18, 29–31]. CTRA has been applied to several different bony lesions including vertebral metastases [29], pediatric patients with benign skeletal lesions [30, 31], and femoral lesions [5, 12] demonstrating a higher specificity with CTRA analysis than what has been historically shown with Mirels for long bone lesions. Two manuscripts assessing fracture risk for femoral lesions directly compared CTRA and Mirels scoring. Nazarian et al. evaluated 104 patients with femur lesions and found the sensitivity of CTRA to be 100% and specificity to be 90%, while Mirels was found to be 71% sensitive and 50% specific [12]. Damron et al. analyzed 94 patients and found CTRA sensitivity and specificity to be 100% and 61%, respectively, while Mirels demonstrated 67% sensitivity and 48% specificity [5]. Interestingly, the same paper analyzed Mirels score cut-offs of 8, 9, and 10 and showed that CTRA was overall superior no matter which value was used as the cut-off. These manuscripts have shown that when using CTRA compared to Mirels scoring, sensitivity for identifying those at risk of fracture remains relatively high, but CTRA has improved specificity. Based on the high sensitivity and specificity of CTRA, this suggests that clinical use of CTRA instead of Mirels scoring may better identify the patients at highest risk of fracture and who would benefit from prophylactic stabilization surgery, with less overtreatment of those who will not go on to fracture.

More recently, a curved beam CTRA approach has been developed to more accurately calculate rigidity for the ends of long bones, such as the femur, given the intrinsic curvature [32]. Traditional CTRA, which was used for the subjects in this study, uses straight beam theory and thus loses accuracy at the proximal and distal ends of long bones. The intertrochanteric region of the proximal femur has been shown to have significant intrinsic curvature and is also a common area for MBD [32]. The curved CTRA model is a promising improvement on the traditional straight CTRA predictions and has been reported as more accurate in predicting the magnitude of failure, as well as the location of failure [13]. These claims are based on a study using fresh frozen cadavers where one femur was used as a control while the other had a simulated lytic defect. While the results appear encouraging, further research is needed to assess the technique clinically.

### 5.3. Finite Element Analysis (FEA)

FEA is the second CT-based tool used for fracture risk assessment in MBD examined in this study. Like CTRA, FEA uses both the 3D geometry and the density of the bone. However, FEA utilizes computer software to create a 3D femur model with lesions from the CT scan set. The model is discretized into small finite elements that are assigned bone strength and stiffness properties using the bone density information [33, 34]. Once the model is created, specific forces are applied to the femoral head and greater trochanter to simulate muscular and joint forces. The applied force is incrementally increased until the femur is predicted to fail [32, 33]. In the current series, the loading conditions included axial compression, level walking, and aggressive stair ascent as previously reported.

Direct comparison of FEA with Mirels illustrates the superiority of FEA. Goodheart et al. [11] compared FEA to the Mirels scoring system, showing a similarly high sensitivity for both tests, but in terms of specificity, FEA was found to be significantly higher. There is limited literature that assesses whether CTRA or FEA is superior in the ability to discriminate cases that fracture from those that do not. Anez-Bustillos et al. conducted a laboratory-based study in which femoral lesions were created in cadaver bones [10]. They found that the correlation between predicted and actual bone strength was not different for CTRA and FEA. Another laboratory-based study by Oftadeh et al. suggested that FEA is more accurate than straight beam CTRA [13], but curved beam analysis produced results that were similar to FEA in terms of predicted strength. In the series we report here, FEA was never used for clinical decision making, as all analyses were done post hoc. Among the cases reported, in only one case, did FEA differ from CTRA, and in that case, the FEA was a false positive.

One of the advantages of FEA over CTRA is that FEA accounts for true loading conditions, and this has been shown to be an important variable in fracture prediction. FEA is able to account for muscular attachment points and forces generated on the femur with both weight-bearing and muscular contraction [11, 32]. Despite the benefits and promising accuracy of FEA, using FEA as a fracture risk prediction tool remains cumbersome due to its complexity, time requirements, cost, and limited availability. FEA requires a trained expert to both build and run the models, and each model requires several hours of staff time to run. CTRA is much more computationally efficient compared to FEA but also requires custom software to generate the rigidity plots. In addition, neither FEA nor CTRA is currently available as plug-in to standard CT software.

Selection of these cases was based upon the CTRA results to illustrate each fracture prediction result (TP, TN, FP, FN) and obviates any conclusion regarding the accuracy of CTRA. However, examples of FP and FN CTRA results were few and far between within this much larger population of patients while TP and TN cases were the norm. This is consistent with the reported high rates of sensitivity and specificity for CTRA [5, 12, 30]. Use of CTRA is limited to only those institutions actively enrolling patients in the ongoing MSTS study and as a research tool is not always available in real time for clinical decision making.

As a pictorial review, this paper provides examples of fracture risk prediction in MBD focusing on CTRA with comparison to Mirels and FEA. Table 2 compares the utility and limitations of the three scoring systems (Mirels, CTRA, FEA) to summarize the technical considerations when using each of these tools. As seen within Table 2, all three scoring systems tend to have a high sensitivity; therefore, the differences in accurate detection lie in the different specificities of these scores. Reports of specificity for the different scoring systems have shown a wide range, but overall the trend is that Mirels has a poor specificity which has been improved with the use of CTRA or FEA. However, this is difficult to translate into practice since Mirels scoring can easily be used by the physician, whereas CTRA and FEA have much higher barriers to access. Mirels scoring can be done simply with an X-ray and some information from the patient. Both CTRA and FEA require CT scans, custom computer software, and people trained in the technique. Among these CT-based techniques, CTRA seems to be more accessible to physicians, whereas FEA is still a technique limited to the lab with minimal clinical utility at this point due to its limitations. No perfect system exists, and further refinement of these CT-based techniques in larger patient populations is needed.

## Figures and Tables

**Table 1:** Case summary based upon CTRA statistical outcome.

Category (based on CTRA)	Case number	Fracture outcome	Mirels	CTRA	FEA
True positives	Case 1	Fracture	10	Increased risk	Increased risk
	Case 2	Fracture	11	Increased risk	Increased risk

False positives	Case 3	No fracture	7	Increased risk	Increased risk
	Case 4	No fracture	9	Increased risk	Increased risk

False negatives	Case 5	Fracture	9	Normal	N/A

True negatives	Case 6	No fracture	8	Normal	Normal
	Case 7 (left femur)	No fracture	8	Normal	Increased risk
	Case 7 (right femur)	No fracture	9	Normal	Normal

**Table 2:** Comparison of Mirels, CTRA, and FEA technical considerations.

Prediction method	Imaging method	Specialized software	Analysis time	Reported sensitivity (%) [16]	Reported specificity (%) [16]	Types of loading	Anatomic/modeling limitations
Mirels	Planar X-ray	No	<5 minutes	71–100	13–94	Not applicable	None

CTRA	Computed tomography (CT)	Yes, to calculate section rigidities	<15 minutes, with custom software	100	60–90	Axial, bending, torsion	Errors associated with the ends of long bones

FEA	Computed tomography (CT)	Yes, to build model and run analysis	2–8 hours, requiring engineering expertise	80–100	63–86	Functional loading (stance, gait, stair climb, etc.)	Models and loading for proximal femur different from distal femur

## Data Availability

Each case report was taken from a larger pool of subjects enrolled in an ongoing Musculoskeletal Tumor Society multicenter study.
